# The influencing factors of participation in time banking volunteer service for older adults among university students in Nanjing, China

**DOI:** 10.3389/fpubh.2023.1289512

**Published:** 2024-01-11

**Authors:** Yue Wu, Siyu Liu, Yating Song, Zhirui Zhang, Yunlong Liu

**Affiliations:** Department of Aging Service and Management, School of Aging Service and Management, Nanjing University of Chinese Medicine, Nanjing, China

**Keywords:** volunteer services for older adults, factor analysis, time banking, theory of planned behavior, sustainable development goals

## Abstract

**Objective:**

This study aims to identify factors influencing university students' participation in time banking volunteer services for older adults and provides evidence to promote the involvement.

**Methods:**

Conducted in November 2022, we utilized a convenience sampling method to recruit students from the School of Aging Service and Management at Nanjing University of Chinese Medicine, China. Data was collected through an online questionnaire focusing on various aspects related to time banking volunteer services for older adults. Factor analysis was employed to extract variables, and logistic regression was applied to identify key determinants.

**Results:**

A significant majority (82.67%) of participants expressed willingness to engage in volunteer services for older adults. Factor analysis uncovered six influential factors explaining 62.55% of the variance. Logistic regression highlighted four key determinants of students' willingness: value judgment (OR = 4.392, CI = 2.897–6.658), social support (OR = 1.262, CI = 0.938–1.975), social influence (OR = 1.777, CI = 1.598–3.799), and socioeconomic conditions (OR = 1.174, CI = 1.891–3.046).

**Conclusion:**

To foster sustainability and continuous time banking among university students majoring in aging service and management, a multifaceted support involving governmental, social, and university is recommended.

## 1 Introduction

Time banking offers a unique way for people in a community to exchange services using time instead of money. In this system, 1 h of any service is equivalent to one time credit. Individuals can join a time bank either through a local group or online, listing both their skills and needs. When a member provides a service, they earn credits, which can be used to receive services from others. Time banks keep track of these credits. Participation is voluntary, allowing members to choose the services they wish to offer and receive (1, 2). With the global older adults population projected to increase from 6.9% in 2000 to 19.3% by 2050, the demand for care services is set to escalate. China has the world's largest older adults population. The 2019 national census data reveals that the population aged 65 and over in China has reached 176 million, representing over one-fifth of the global population in this age group. This large and rapidly increasing older adults population poses significant economic and social challenges for China. Time banking encourages younger individuals to invest their time in various community services, earning credits that they can redeem for their own future needs. This approach can relieve the pressure on existing care services by facilitating informal social support and engaging socially excluded groups in community activities, thereby promoting social inclusion (3). Furthermore, time banking fosters an alternative, community-focused economic model where participants both contribute and benefit. This system highlights the value of collective efforts and a community-based lifestyle, achieved through the exchange of time and expertise (4). Such practices enhance the sense of community involvement, particularly benefiting newcomers or those living in isolation (5). Feeling connected, a key aspect of social capital, is crucial in improving overall wellbeing. Time banking proves particularly beneficial for the older adults, low-income individuals, and those living alone, as it boosts both physical health and a sense of belonging to a community (6–8). The China Time Bank Development Research Report indicates that time banking is gaining increased attention within the Chinese academic community. However, its expansion is impeded by several factors, including limited understanding of its value, inadequate institutional frameworks, and operational management support. This study aims to provide evidence supporting the involvement of time banking in care services for older adults.

In adherence to Sustainable Development Goals (SDG) 3, “Good Health and Wellbeing,” and SDG 10, “Reduced Inequalities,” special consideration is necessary for the older adults—a group often encountering restricted care access and ageism, both factors that can substantially impair their wellbeing (9, 10). The integration of home and community-based care models is advocated to improve outcomes for older adults (11). The drive toward establishment of age-friendly communities, which emphasize the welfare and quality of life for the older adults, resonates with a global aspiration (12). Time banking services not only enhance access to care for older adults but also counter potential disparities and discriminatory practices, fostering age-friendly environments. A key action area for such communities includes bolstering community support, foundational for healthy aging and aimed at elevating wellbeing and life satisfaction (13). Evidence shows that involvement in time banking can cultivate trust and promote goodwill within schools and urban areas (14), and it has been recommended as a method to harness social capital to counteract the challenges associated with an aging population (15). The 2021 Time Bank Development Research Report in China underscores the significance of incorporating time banking into higher education to enrich students' ethical awareness and practical abilities through volunteerism (16). Moreover, we posit that volunteering, traditionally viewed as an altruistic activity, may also be significantly shaped by diverse socio-economic factors (17). The aim of this study is to explore the determinants that influence university students' participation in time banking services and to amass evidence to bolster student engagement.

In China, time banking volunteer service for older adults among university students exhibit regional diversity. In Hunan Province, the Time Bank of Wangxianqiao Community has established a symbiotic relationship with neighboring colleges in Changsha County. This initiative transforms the community into a practical base for student volunteering and integrates these activities into the moral education system. Meanwhile, in Henan Province, the Youfang Community's Time Bank, developed in collaboration with the Zhishan Social Work Service Center of Henan University, showcases a partnership between social work centers and communities, with the project specifically designed to facilitate student social practice services. In Zhejiang Province, the Green Kang Time Bank demonstrates the cooperative efforts between higher education institutions, such as Zhejiang Gongshang University and Zhejiang Sci-Tech University, to serve the older adults through the engagement of college students. This collaboration not only enriches students' moral and practical experience but also grants official recognition of their volunteer services. Additionally, the School of Aging Service and Management at Nanjing University of Chinese Medicine (NJUCM), the first in China offering undergraduate programs in this field since 2020, represents a significant academic initiative. Endorsed by the Ministry of Civil Affairs, this school is at the forefront of the national active aging strategy, emphasizing the traditional ethos of respecting the older adults and promoting professional ethics in care for older adults through time-banking services. With a focus on this university community time banking, this study aims to explore the typical operation mode of China's time bank among youth groups.

## 2 Theoretical background

This research is grounded in the Theory of Planned Behavior (TPB), which posits that individual behavior is driven by behavioral intentions where these intentions are a function of an individual's attitude toward the behavior, the subjective norms surrounding the performance of the behavior, and the perceived behavioral control over the behavior (18). TPB has been widely applied to studies of volunteerism and is particularly relevant to understanding the motivations, a domain where volunteer services are exchanged within a community (19, 20). While TPB provided a valuable theoretical foundation for our research, we recognized certain limitations in its ability to fully account for the phenomena observed in time banking, particularly in the context of volunteer services for older adults. Literature highlights trust and social support as central to participation in time banking (21, 22). Trust, both knowledge-based and swift, is identified as crucial for the sharing of resources and successful collaboration within time banks (23, 24). Social cognitive theories further elucidate the psychological processes behind such community engagements (25). We adopted an exploratory approach to broaden our investigation beyond the confines of TPB. This approach allowed us to incorporate additional variables related to social support, and specific cultural factors that are crucial in the context of Chinese older adults' care. Drawing from the core principles of TPB, we integrated variables related to attitudes toward volunteer services for older adults, subjective norms, perceived behavioral control, and intentions associated with these volunteer services, as indicated by existing studies (21, 26, 27). Additionally, incorporated investigation that delve into the students' socioeconomic characteristics and their attitudes toward traditional older adults' care culture in China, such as “filial piety” (28). By integrating these supplementary aspects with the core TPB framework, our study aimed to provide a more comprehensive understanding of the factors influencing students' engagement in time banking. We meticulously selected a comprehensive range of variables for survey, applying factor analysis to derive actionable insights that inform the practical implementation of time banking practices in universities.

## 3 Data and methods

The questionnaire was distributed online, with data collection taking place in November, 2022. A convenience sampling method recruited students from the School of Aging Service and Management at NJUCM, China. A total of 374 students participates in the survey. Out of these, 254 responses contained no missing values, and did not present inconsistent information were included in the final analysis. We adhered strictly to the ethical standards associated with social surveys in our study. All participants provided informed consent, and we took meticulous care to explain the nature and purpose of the study, ensuring a comprehensive understanding before participation. Collected data is anonymized and handled with utmost care to prevent unauthorized access. Additionally, our research is part of projects funded by the school (funding numbers: 2023YLFWYGL006 and 2023YLFWYGL014). The projects have received formal approval and were signed off through the “Research Integrity Commitment Letter” from NJUCM, signed by the Dean and attests to our commitment to upholding research integrity, ethical standards, and compliance with all relevant regulations.

Factor analysis was performed to establish the validity and reliability of the influencing factors of participation in time banking volunteer services for older adults in this study. First, Bartlett's test of sphericity and the Kaiser–Meyer–Olkin (KMO) test were used to examine whether the correlation matrix was an identity matrix, thereby verifying the independence of all the variables included and assessing the suitability of the dataset for factor analysis. Based on the eigenvalues, the number of factors to be extracted was determined, followed by factor rotation to facilitate more straightforward interpretation and understanding of each factor. Furthermore, a logistic regression was conducted to analyze the factors affecting the willingness to participate. The factor scores (which can be obtained directly through STATA operations) were used as independent variables, while the dependent variable was coded as the willingness to participate (with a value of 0 indicating no willingness and a value of 1 indicating willingness). *P* < 0.05 was set as the standard for statistical significance, and STATA 15 software was utilized for the cleaning and preprocessing of the collected raw data.

## 4 Results

### 4.1 Samples characteristics

Table 1 displays the results of a survey on university students' willingness to engage in volunteer services for the older adults, segmented by demographic characteristics. Out of the 254 students surveyed, 210 (82.68%) indicated they were willing to volunteer. The data suggest that students who are female, in their freshman year, enrolled in aging-related majors, from rural backgrounds, or from households with moderate incomes show a greater propensity to volunteer in older adults' care services.

**Table 1:** Sample demographic characteristics (%).

**Variables**	**Groups**	***N***	**Will to participate**		**χ^2^**	***P***
			***N***	**%**		
Gender	Male	72	58	80.56	0.32	0.574
	Female	182	152	83.52		
Grade	Freshman	184	159	86.41	6.51	0.011
	Senior	70	51	72.86		
Major	Aging -related	103	89	86.41	1.68	0.194
	Non- aging-related	151	121	80.13		
Urban-rural	Urban	153	122	79.74	2.32	0.128
	Rural	101	88	87.13		
Household income status	Good	65	51	78.46	1.73	0.422
	Centered	155	132	85.16		
	Poor	34	27	79.41		

Table 2 shows the attitudes, subjective norms, perceived behavioral control, and intentions, along with attitudes toward traditional older adults' care culture among the investigated university students and their willingness to engage in time banking volunteer services for the older adults. Behavioral attitude dimension indicates a strong agreement with the benefits of time banking, such as happiness, satisfaction, skill enhancement, and social experience accumulation, with high percentages of students willing to participate ranging from 87.5% to 92.89%. Subjective norms aspect shows high percentages of willingness to participate were also observed among students who felt support from parents, peers, and the university, or who saw high mobilization and trustworthiness in such activities. Perceived behavior control dimension shows that students who perceive themselves as having a good physical condition, adequate knowledge, skills, expertise, and leisure time, and who find the activities conveniently located, are more inclined to participate. A significant majority of students would tell their friends about becoming a member of a time bank. Lastly, attitudes toward traditional older adults' care culture indicates that those who feel a sense of guilt for not accompanying older adults or who are willing to communicate with older adult strangers have a higher percentage of willingness to participate in time banking.

**Table 2:** Attitudes and factors of willingness to participate in time banking.

**Variables**	**Groups**	***N***	**Will to participate**		**χ^2^**	***P***
			***N***	**%**		
**Behavioral attitude**						
Participating in time banking can make me happy	Disagree	43	14	32.56	90.79	<0.001
	Agree	211	196	92.89		
Participating in time banking can make me feel satisfied	Disagree	38	10	26.32	99.11	<0.001
	Agree	216	200	92.59		
Participating in time banking can enrich my leisure time	Disagree	37	10	27.03	93.65	<0.001
	Agree	217	200	92.17		
Participating in time banking can help me make more friends	Disagree	46	20	43.48	60.27	<0.001
	Agree	208	190	91.35		
Participating in time banking can help me enhance my skills	Disagree	23	6	26.09	56.55	<0.001
	Agree	231	204	88.31		
Participating in time banking can help me accumulate social experience	Disagree	22	7	31.82	43.5	<0.001
	Agree	232	203	87.5		
Participating in time banking can help me realize my self-worth	Disagree	37	9	24.32	102.97	<0.001
	Agree	217	201	92.63		
Participating in time banking can help me solve social problems	Disagree	49	23	46.94	54.14	<0.001
	Agree	205	187	91.22		
**Subjective norms**						
The degree of parents' support of participation in time banking activities	Low or neutral	87	57	65.52	27.21	<0.001
	High	167	153	91.62		
The degree of peer recognition for participating in time banking activities	Low or neutral	89	62	69.66	16.2	<0.001
	High	165	148	89.7		
The degree of the university's recognition for participating in time banking activities	Low or neutral	63	39	61.9	25.24	<0.001
	High	191	171	89.53		
Mobilization level of time banking activities	Low or neutral	92	59	64.13	34.64	<0.001
	High	162	151	93.21		
Trustworthiness level of time banking activities	Distrust or neutral	41	16	39.02	65.05	<0.001
	Trust	213	194	91.08		
The degree of exemplary participation by public figures and role models in time banking activities	Low or neutral	80	50	62.5	33.2	<0.001
	High	174	160	91.95		
The degree of guidance I receive from moral beliefs	Low or neutral	51	22	43.14	69.66	<0.001
	High	203	188	92.61		
The degree of influence of public opinion on me	Low or neutral	89	59	66.29	25.68	<0.001
	High	165	151	91.52		
My physical condition	Poor or neutral	58	38	65.52	15.45	<0.001
	Good	196	172	87.76		
My knowledge, skill and expertise	Poor or neutral	80	51	63.75	29.21	<0.001
	Good	174	159	91.38		
My leisure time	Less or neutral	85	66	77.65	2.26	0.133
	More	169	144	85.21		
The location of the time banking activities	Poor or neutral	89	67	75.28	5.23	0.022
	Good	165	143	86.67		
Perceived personal responsibility risk	Less or neutral	108	79	73.15	11.91	0.001
	More	146	131	89.73		
The convenience of participating in activities	Poor or neutral	95	75	78.95	1.47	0.225
	Good	159	135	84.91		
The opportunity to participate in activities	Poor or neutral	96	65	67.71	24.14	<0.001
	Good	158	145	91.77		
**Behavioral intentions**						
I will tell my friends about becoming a member of a time bank	Disagree	35	14	40	51.62	<0.001
	Agree	219	196	89.5		
**Attitudes toward traditional older adults' care culture**						
I feel guilty if I do not accompany older adults in my family for a long period of time	Disagree	56	30	53.57	42.49	<0.001
	Agree	198	180	90.91		
I am willing to communicate with older adult strangers	Disagree	79	45	56.96	52.94	<0.001
	Agree	175	165	94.29		
I often express gratitude for the hard work and dedication exhibited older adults in my family	Disagree	24	6	25	61.56	<0.001

### 4.2 Multicollinearity analysis

The multicollinearity of the factors influencing participation willingness was assessed using Bartlett's test of sphericity and the Kaiser–Meyer–Olkin (KMO) measure of sampling adequacy. Bartlett's test of sphericity tests the assumption that the correlation matrix is an identity matrix. This test yielded a result of χ^2^ = 3736.279 (*P* < 0.001), leading to the rejection of the null hypothesis. The KMO statistic measures the degree of shared variance among variables by comparing the magnitudes of simple correlation coefficients to partial correlation coefficients. A KMO value of 0.871 was obtained, thus implying a substantial overlap of information among the variables and thereby indicating their suitability for factor analysis. Given these results, proceeding with factor analysis and multiple regression analysis was deemed appropriate.

### 4.3 Factor analysis

The results of the factor analysis are shown in Table 3. According to the criterion of an eigenvalue ≥1, 6 factors were extracted, which exhibited a cumulative contribution rate of 62.55%. After the maximum variance orthogonal rotation, the rotated component matrix was obtained, as shown in Table 4.

**Table 3:** Factor analysis results.

**Factor**	**Eigenvalue**	**Difference**	**Contribution (%)**	**Cumulative contribution (%)**
F1	8.38284	5.5239	0.2994	0.2994
F2	2.85895	0.54399	0.1021	0.4015
F3	2.31496	0.62229	0.0827	0.4842
F4	1.69267	0.47031	0.0605	0.5446
F5	1.22236	0.17969	0.0437	0.5883
F6	1.04267	0.05931	0.0372	0.6255
F7	0.98336	0.03907	0.0351	0.6606
F8	0.94429	0.02211	0.0337	0.6944
F9	0.92218	0.08972	0.0329	0.7273
F10	0.83246	0.13373	0.0297	0.757
F11	0.69873	0.06892	0.025	0.782
F12	0.62981	0.06152	0.0225	0.8045
F13	0.56829	0.02151	0.0203	0.8248
F14	0.54677	0.01055	0.0195	0.8443
F15	0.53622	0.02894	0.0192	0.8634
F16	0.50728	0.09497	0.0181	0.8816
F17	0.41231	0.00299	0.0147	0.8963
F18	0.40932	0.02105	0.0146	0.9109
F19	0.38826	0.04355	0.0139	0.9248
F20	0.34471	0.02443	0.0123	0.9371
F21	0.32029	0.04133	0.0114	0.9485
F22	0.27896	0.0128	0.01	0.9585
F23	0.26616	0.00991	0.0095	0.968
F24	0.25625	0.02166	0.0092	0.9771
F25	0.23459	0.03326	0.0084	0.9855
F26	0.20133	0.08133	0.0072	0.9927
F27	0.12	0.03603	0.0043	0.997
F28	0.08398	.	0.003	1

**Table 4:** Component matrix after factor rotation.

**Variables**	**Factors**					
	**F1**	**F2**	**F3**	**F4**	**F5**	**F6**
Participating in time banking can help me realize my self-worth	0.8638	0.141	0.0792	0.1252	0.0278	−0.0498
Participating in time banking can help me enhance my skills	0.8621	0.1187	0.1473	−0.0108	−0.0563	0.0012
Participating in time banking can make me feel satisfied	0.8613	0.0739	0.0668	0.2396	0.0106	0.0084
Participating in time banking can help me accumulate social experience	0.8554	0.1201	0.1256	−0.0689	−0.0694	−0.0485
Participating in time banking can enrich my leisure time	0.8462	0.0396	0.0553	0.1658	0.0347	0.0683
Participating in time banking can make me happy	0.7717	0.0806	0.0629	0.32	−0.0037	0.0001
Participating in time banking can help me make more friends	0.7617	0.0704	0.1353	0.1087	−0.0484	0.0411
Participating in time banking can help me solve social problems	0.7376	0.0715	0.0816	0.1519	0.0575	−0.1953
The degree of university's recognition of participation in time banking activities	0.1546	0.8404	0.0362	0.1378	0.0998	−0.1397
The degree of peer recognition of participation in time banking activities	0.1338	0.797	0.0619	0.1866	−0.0817	0.105
The degree of parents' support of participation in time banking activities	0.1587	0.7687	−0.0728	0.3032	−0.1118	0.127
The convenience of participating in activities	0.1246	0.0958	0.729	−0.0073	−0.1797	0.0027
My leisure time	0.0917	−0.2135	0.7037	0.0916	0.084	−0.0793
The location of the activities	0.1532	0.0813	0.6994	0.0037	0.0109	0.1039
Perceived personal responsibility risk	0.2203	0.0375	0.639	−0.0921	0.0233	0.101
The opportunity to participate in activities	0.3007	0.0373	0.6335	0.1762	0.0125	−0.1109
The degree of exemplary participation by public figures and role models in time banking activities	0.2485	0.4024	−0.0084	0.655	−0.0304	−0.0529
The degree of influence of public opinion on me	0.1552	0.4344	0.0321	0.6424	−0.0476	0.02
The degree of guidance I receive from moral beliefs	0.4199	0.1778	0.016	0.6393	0.1408	0.0081
Trustworthiness level of time banking activities	0.4625	0.2471	0.0885	0.5842	−0.0283	−0.0616
Mobilization level of time banking activities	0.2332	0.3933	0.1368	0.5799	0.0709	−0.1365
Household income status	−0.0084	−0.0235	−0.0127	0.01	0.8174	0.1535
Urban–rural	−0.0174	−0.1699	−0.1091	0.139	0.7173	−0.1296
Grade	−0.1617	−0.1381	−0.0151	0.0648	−0.1308	0.646
Gender	0.0116	−0.14	−0.0395	0.1698	−0.2128	−0.6847
Physical condition	0.1942	0.1938	0.36	0.2396	−0.1037	0.3052
Knowledge and skill expertise	0.0884	0.2625	0.3818	0.3681	−0.0008	0.2605
Major	0.0341	−0.2496	−0.1128	0.2101	−0.5776	0.0549

F1: Value Judgment Factor. This factor encompasses perceptions of volunteer services for older adults as avenues for personal growth and satisfaction. Specifically, it includes elements such as realizing one's self-worth, exercising one's abilities, deriving satisfaction, accumulating social experiences, enriching leisure time, fostering happiness, facilitating friendships, and contributing to social solutions.

F2: Social Support Factor. Recognition from key social relations, including schools, peers, and parents, is included as part of this factor, which emphasizes the role of external validation and encouragement.

F3: Objective Conditions Factor. This factor considers the tangible elements affecting participation, such as convenience, the availability of free time, the location of the activity, potential personal responsibility risks, and participation opportunities.

F4: Social Influence Factor. Aspects such as role modeling by public figures, prevailing social opinion, guidance from moral beliefs, the trustworthiness of volunteer activity organizers, and participant mobilization efforts are included under this classification.

F5: Socioeconomic Factor. Components pertaining to an individual's background, including his or her family's economic status and urban or rural residence, constitute this factor.

F6: Knowledge and Skills Factor. This final factor contains elements that are linked to one's professional profile, including the individual's grade, knowledge and skill expertise, major, and other professional competencies. It also integrates gender, physical condition, and other elements that influence an individual's self-assessment of their own knowledge and capabilities.

### 4.4 Multivariate logistic regression analysis

A multivariate logistic regression was conducted to explore the influence of the previously identified factors on undergraduates' willingness to participate in volunteer services for older adults. The results of a chi-square goodness-of-fit test of the model yielded a value of 110.64 (*P* < 0.001), thus emphasizing the model's robustness. Table 5 illustrates the detailed logistic regression outcomes: the Value Judgment Factor (F1) exhibited a strong positive association with students' willingness to participate, with the highest odds ratio (OR = 4.39, *P* < 0.001). The Social Support Factor (F2), Social Influence Factor (F4), and Socioeconomic Factor (F5) also significantly encouraged participation (OR > 1, *P* < 0.001). The Objective Conditions Factor (F3) exhibited a trend toward promoting participation, although this impact did not achieve statistical significance (*P* > 0.05). The Knowledge and Skills Factor (F6) seemed to potentially discourage participation, with an OR of 0.918 (*P* > 0.05).

**Table 5:** Logistic regression of the factors affecting willingness to participate in elder care volunteer services.

**Factors**	**O R**	**S. E**.	**z**	***P* > z**	**95% Conf**.	**Interval**
F1 (Value judgment factor)	4.392	0.932	6.970	0.000	2.897	6.658
F2 (Social support factor)	1.938	0.424	3.020	0.002	1.262	2.975
F3 (Objective conditions factor)	1.395	0.331	1.400	0.161	0.876	2.221
F4 (Social influence factor)	2.598	0.503	4.930	0.000	1.777	3.799
F5 (Socioeconomic factor)	1.891	0.460	2.620	0.009	1.174	3.046
F6 (Knowledge and Skills Factor)	0.918	0.209	−0.380	0.708	0.588	1.434

## 5 Discussion

To alleviate pressures from an aging population, time banking emerges as a promising strategy. It dovetails with the objectives of Sustainable Development Goals 3 and 10, particularly in prioritizing support for older adults as a vulnerable demographic. Despite its potential, Time banking is not widespread in China. One of the primary challenges in the widespread adoption of time banking in China is rooted in the cultural context. Traditional Chinese values emphasize family-based care for the older adults. This may limit the perceived need or acceptance of alternative systems like time banking, which are based on community sharing and reciprocity. Meanwhile, there is a relative lack of awareness and understanding of the time banking concept among the general population in China. Time banking, as a relatively new and Western-origin concept, is not widely recognized. Moreover, the policy environment in China has not yet fully adapted. It was only toward the end of 2021 that the term “time bank” was officially incorporated into a government document - the “Beijing Elderly Care Services Time Bank Implementation Plan (Trial).” In 2022, the development of the “time bank” mutual aid model for older adults' was explicitly proposed as a task in the Beijing government work report during the “Two Sessions.” However, this concept has not yet been implemented nationwide. The absence of comprehensive government endorsement may also constitute a barrier, impacting public trust and legitimacy. Besides, the development of time banking requires a robust technological infrastructure to facilitate the exchange of services and time credits. Especially in rural or less developed regions of China, the necessary technological infrastructure is not sufficiently advanced to support such systems. The School of Aging Service and Management at NJUCM, offering an undergraduate degree in aging service and management, reflects a Chinese governmental strategy to address the aging challenge (29). Our research based on this, probes the variables that affect university students' willingness to engage in time banking volunteer services for older adults. The aim is to uncover practical, data-driven strategies that can enhance the adoption and efficacy of time banking in universities.

Firstly, we found that 82.67% of the students in this survey expressed their willingness to participate in the provision of volunteer services for older adults, and students who are female, in their freshman year, enrolled in aging-related majors, from rural backgrounds, or from households with moderate incomes show a greater propensity to volunteer in care service for older adults. While students exhibit a substantial inclination toward participation in time banking volunteer services for older adults, a pronounced discrepancy exists between this intent and their actual participation. Such an inconsistency is in line with the findings of previous research (30). Several factors could contribute to it. Lack of awareness or access to opportunities, students may be willing to participate but are not aware of available opportunities or face barriers in accessing them. Moreover, there may be a difference between general willingness and the motivation required to take concrete action. Such as perceived impact, personal gains, and social recognition play a role in transitioning from intent to action. This gap between intent and participation is an important area for future research. Studies specifically designed to investigate these barriers and facilitators to participation would provide valuable insights into converting willingness into active engagement.

Among them, female students exhibit higher participatory willingness, which can potentially be attributed to the perceived traits of patience and empathy (31). The motivations for volunteering across different gender groups, additional evidence is needed to substantiate this analysis. Freshmen, who are new enrollees, might experience less study pressure and thus have more time and enthusiasm for volunteerism. Students in aging-related majors, due to their study focus, likely possess deeper insights into the needs of and challenges faced by older adult individuals, thereby exhibiting an increased propensity to serve this demographic. The tight-knit familial and community bonds observed in rural areas might foster a heightened sense of community service among these students (32). Students from moderate economic backgrounds, compared to their more affluent or economically disadvantaged peers, may have more time and resources to dedicate to volunteer activities (33). This could potentially lead to a stronger and more sustained willingness to participate. The particularities of volunteer services for older adults in the context of Chinese traditional culture, contrasted with the “volunteerism elitism” perspective prevalent in the West counties (34), offer a fresh perspective in understanding volunteer behavior in care for older adults within the unique Chinese context. It is noteworthy that groups showing less willingness to engage in voluntary services could be at a heightened risk of social exclusion. This is because volunteering is often perceived not only as a means to augment individual resources but also as a vital pathway for reducing social exclusion. It facilitates “creating avenues for reintegration into society and provides opportunities for individuals to become more involved in their communities” (35).

Secondly, the research indicates that several factors influence university students' willingness to participate in time banking care services. A predominant factor is their value judgments. Students place a high value on the intrinsic benefits they derive from participation, such as self-realization, skill development, satisfaction, and enriching social experiences. This observation is consistent with a longitudinal study of first-time non-violent juvenile offenders in Washington, DC. Participants referred to the Youth Court showed the effectiveness of voluntary service programs (36). A significant correlation was found with the development of life skills, particularly in areas like goal setting, problem-solving, decision-making, and academic learning (37). In another study, an intervention framework for co-production and time banking, designed to assist practitioners in implementing time banking projects, revealed an interesting trend. Initially, student participation was involuntary, but it evolved into a semi-voluntary engagement. This transition was marked by a significant increase in emotional and cognitive engagement, leading to the development of social skills, improved self-esteem, and positive identity formation (38). Furthermore, trust theory suggests that when students believe their participation will fulfill the intrinsic values mentioned earlier, their commitment tends to become more steadfast (39). A parallel can be drawn with a survey on older adults' motivations for sustained volunteerism, where the primary driving force was societal contribution, deeply rooted in a spirit of altruism (40). Given these insights, it becomes crucial for universities and government bodies to tailor time banking and volunteer services for older adults to align with these motivations and needs. Such alignment not only meets the specific requirements of the older adults but also enhances the engagement and fulfillment of the student volunteers.

Our findings also reveal that the social support factor, including recognition from key social relations such as schools, peers, and parents, plays a significant role. This factor is crucial according to the theory of social capital, which posits that social relations and networks profoundly influence individual behaviors. They provide individuals with support, resources, and pertinent information, thereby increasing their likelihood of engaging in volunteer services for older adults (41). Conversely, time banking contributes significantly to the development of social capital (15). Participants in time banking, who exhibit increased self-efficacy, also demonstrate a heightened sense of community (42, 43). Additionally, we discovered that the influence of social influence, public figures, and public opinion is significant. Public figures' endorsements or criticisms can not only shape public opinion but also enhance or diminish the perceived credibility of social causes and initiatives (44). Celebrities and influential personalities can inspire and motivate students by setting an example through their own involvement or endorsement. Public opinion, shaped by media and social discourse, raises awareness about the importance of such volunteering, potentially making it a socially valued and expected activity. This creates a conductive environment where university students feel encouraged and motivated to participate, aligning their actions with societal expectations and the examples set by admired figures (17, 45). Moreover, social cognitive theory suggests that individual behavior is influenced by social cognitive processes (46, 47), this influence is mediated by established social norms, collective identity, and channels of information dissemination, thereby fostering individual involvement in volunteer services for older adults (48). Observing peers, relatives, or community members engage in volunteer services for older adults, an individual might perceive such behavior as socially approved and valuable, thus influencing their own behavior. This type of influence, achieved through social norms, group identity, and information transmission mechanisms, encourages participation (49). In addition to these, factors related to knowledge and skills may also influence individuals' willingness to participate in volunteer services for older adults (50). Lastly and in line with previous studies (51, 52), socioeconomic factors may influence individuals' willingness to participate in volunteer services. This highlights the need for policy-makers to consider students' actual conditions and avoid excessive motivation.

While the findings show that volunteer projects organized by educational institutions are influenced by a variety of factors, there are concerns about their long-term viability. The sustainability and ongoing commitment of students to community service pose significant challenges in China, particularly in Hong Kong (15). For example, secondary school students are typically engaged in the Other Learning Experience (OLE) project, which relates to community service, often are unable to develop a strong sense of personal and social responsibility within their communities (53). This implies that despite previous experiences, their future participation in volunteer services may not be sustained. The motivational factors influencing university students may significantly differ, given their increased autonomy and more defined career and social responsibility perspectives. As highlighted in the China Time Bank Development Research Report, time banking is increasingly gaining attention in the Chinese academic sphere. However, its prolonged development faces challenges, including a limited general understanding of its benefits, and the need for more robust institutional frameworks and operational management support. To foster sustainable and continuous engagement in time banking among university students, a synergistic approach involving government, society, and educational institutions is crucial. In light of our study's insights, we propose a comprehensive strategy for promotion that includes: Firstly, further popularizing the concept of time banking in China is crucial. The Government should provide more resources in the community and subsidies for universities, including raising awareness about the value of time banking, which aligns with the SDGs for improving older adults' access to care and addressing issues of limited opportunities and discrimination. Additionally, coordinating the activities for the exchange of time and service is vital. Given that time banks often struggle to cover administrative costs, government and university financial support can aid both promotion and operations of time banks, helping university students perceive such behavior as socially approved and valuable. Moreover, social commercial organizations, non-governmental organizations (NGOs), and family units can also play a significant role in the promotional process of time banking. Furthermore, Party members in China, who are pivotal in public welfare activities (2), therefore could encourage the student members to participate as the exemplary deeds.

This study focuses on identifying factors influencing university students' participation in time banking volunteer services for older adults and providing evidence to promote involvement, which is in line with university education of major in aging service and management in China, emphasizes traditional cultural values, mirroring the “virtue-first” approach and the respect for the older adults deeply ingrained in Chinese society. These principles are akin to the concepts of “character education” or “moral education” prevalent in Western countries, aimed at nurturing well-rounded value systems and moral judgment in students. As the related undergraduate programs become more widespread in Chinese universities, our research provides essential insights for practical volunteer service education in these areas. Moreover, the application of our findings in promoting time banking services among other youth groups in China could serve as a model of reference with time banking volunteer services for older adults among university students exhibit regional diversity.

We acknowledge key limitations in our research and suggest directions for future studies. Our use of an exploratory survey design is a crucial factor to consider while interpreting our results. One specific limitation is the focus on NJUCM, known for its emphasis on traditional Chinese cultural education, which might influence the higher willingness among surveyed students to participate in volunteer services for older adults. Furthermore, in the data processing phase, there may also be biases such as merging groups with very few samples, and the exclusion of certain variables, despite we based on a theoretical framework. Self-report bias is another limitation that cannot be entirely eliminated in our study. Future research employing diverse methodologies, such as observational studies or third-party reported data, would be valuable to gain a more comprehensive understanding of students' true willingness to volunteer. Additionally, the potential for non-response bias we suggest future studies might explore this further, possibly through more targeted outreach to non-respondents or employing alternative data collection methods.

## 6 Conclusion

We found a strong willingness among university students majoring in aging service and management students to participate in time banking. A multifaceted support involving governmental, social, and university is recommended to promote the involvement.

## Data availability statement

The raw data supporting the conclusions of this article will be made available by the authors, without undue reservation.

## Author contributions

YW: Conceptualization, Data curation, Formal analysis, Methodology, Software, Supervision, Writing—original draft, Writing—review & editing, Validation, Visualization. SL: Data curation, Investigation, Writing—original draft. YS: Investigation, Writing—original draft. ZZ: Investigation, Writing—original draft. YL: Funding acquisition, Project administration, Resources, Writing—review & editing.
